# Weakly Youths

**Published:** 1877-05

**Authors:** 


### WEAKLY YOUTHS.

Within one week, three persons have complained that their lives have been made lives of suffering, by the ignorance of parents, thus: They grew up rapidly, almost as tall at sixteen as at mature age. The rapidity of their growth was attended with great debility, while the parents judging of the ability to work by the size, required more of them than they were able to perform, and a strain was imposed upon their constitutions, which made them a wreck after; not indeed destroying life, but leaving the body a shell, and all its functions so impaired, as to their capabilities, that none of their work was well performed, resulting in disease of the whole system, making life a torture, and in one case we know of, there is a never failing reprehension of parental memory.

Persons who are healthy and hearty themselves, do not know how to sympathize with a rapidly growing child, and their complaints of weariness are unheeded, blamed or scolded at. To all parents then, especially to farmers and mechanics, we give the advice, when a child has grown up rapidly, impose but little labor, and that, never violent nor long protracted; it should be light, short, steady, not by fits and starts, never drive, always encourage, and when they go to bed at a regular, early hour, let them have all the sleep they will take, never allow them to be waked up, let nature do that, and she will do it regularly, and in due time.

